# [Retracted] MicroRNA‑19a‑3p inhibits the cellular proliferation and invasion of non‑small cell lung cancer by downregulating UBAP2L

**DOI:** 10.3892/etm.2022.11557

**Published:** 2022-08-09

**Authors:** Yuejiang Pan, Ke Jin, Xuan Xie, Kexi Wang, Huizhong Zhang

Exp Ther Med 20:2252–2261, 2020; DOI: 10.3892/etm.2020.8926

Following the publication of this paper, it was drawn to the Editors’ attention by a concerned reader that certain of the cell colony formation assay data shown in Fig. 2C, cell invasion assay data in Fig. 3B and Transwell Matrigel invasion assay data in Fig. 6D were strikingly similar to data appearing in different form in other articles by different authors. Owing to the fact that the contentious data in the above article had already been published elsewhere prior to its submission to *Experimental and Therapeutic Medicine,* the Editor has decided that this paper should be retracted from the Journal. After having been in contact with the authors, they agreed with the decision to retract the paper. The Editor apologizes to the readership for any inconvenience caused.

